# Event-related brain-oscillatory and ex-Gaussian markers of remission and persistence of ADHD

**DOI:** 10.1017/S0033291720002056

**Published:** 2022-01

**Authors:** Isabella Vainieri, Giorgia Michelini, Nicoletta Adamo, Celeste H. M. Cheung, Philip Asherson, Jonna Kuntsi

**Affiliations:** 1 Social, Genetic and Developmental Psychiatry Centre, Institute of Psychiatry, Psychology and Neuroscience, King's College London, London, UK; 2 Semel Institute for Neuroscience & Human Behavior, University of California Los Angeles, 760 Westwood Plaza, Los Angeles, California, USA; 3 Education Endowment Foundation, London, UK

**Keywords:** ADHD, ex-Gaussian, brain oscillations, remission

## Abstract

**Background:**

Attention-deficit/hyperactivity disorder (ADHD) often persists into adolescence and adulthood, but the processes underlying persistence and remission remain poorly understood. We previously found that reaction time variability and event-related potentials of preparation-vigilance processes were impaired in ADHD persisters and represented markers of remission, as ADHD remitters were indistinguishable from controls but differed from persisters. Here, we aimed to further clarify the nature of the cognitive-neurophysiological impairments in ADHD and of markers of remission by examining the finer-grained ex-Gaussian reaction-time distribution and electroencephalographic (EEG) brain-oscillatory measures in ADHD persisters, remitters and controls.

**Methods:**

A total of 110 adolescents and young adults with childhood ADHD (87 persisters, 23 remitters) and 169 age-matched controls were compared on ex-Gaussian (mu, sigma, tau) indices and time-frequency EEG measures of power and phase consistency from a reaction-time task with slow-unrewarded baseline and fast-incentive conditions (‘Fast task’).

**Results:**

Compared to controls, ADHD persisters showed significantly greater mu, sigma, tau, and lower theta power and phase consistency across conditions. Relative to ADHD persisters, remitters showed significantly lower tau and theta power and phase consistency across conditions, as well as lower mu in the fast-incentive condition, with no difference in the baseline condition. Remitters did not significantly differ from controls on any measure.

**Conclusions:**

We found widespread impairments in ADHD persisters in reaction-time distribution and brain-oscillatory measures. Event-related theta power, theta phase consistency and tau across conditions, as well as mu in the more engaging fast-incentive condition, emerged as novel markers of ADHD remission, potentially representing compensatory mechanisms in individuals with remitted ADHD.

## Introduction

Attention-deficit/hyperactivity disorder (ADHD) often persists into adolescence and adulthood (Cheung et al., 2016; Faraone, Biederman, & Mick, 2006) and leads to several detrimental outcomes (Asherson, Buitelaar, Faraone, & Rohde, 2016). Identifying the processes underlying ADHD persistence and remission has the potential to inform the development of novel interventions to promote clinical improvement in individuals with persistent ADHD.

Longitudinal studies show that cognitive and neural impairments linked to ADHD, encompassing both higher-level executive processes (e.g. inhibition, working memory) and lower-level processes [e.g. attentional lapses measured by reaction-time variability (RTV)], tend to remain impaired in individuals whose ADHD persist (‘persisters’) (Franke et al., 2018). Fewer studies have examined how individuals who remit from the disorder (ADHD ‘remitters’) compare at the cognitive and neural levels to ADHD persisters and controls. The majority of studies to date report that most executive-functioning impairments do not distinguish ADHD remitters from persisters (Agnew-Blais et al., 2019; Franke et al., 2018), indicating that they may not be sensitive to ADHD remission. In a follow-up study of adolescents and young adults with childhood ADHD, we recently observed that cognitive-electroencephalography (EEG) measures of preparation-vigilance processes were impaired in ADHD persisters compared to remitters and controls, but comparable between remitters and controls (Cheung et al., 2016; James et al., 2017; Michelini et al., 2016). Many of these measures also showed continuous associations with ADHD severity within individuals with childhood ADHD, indicating that preparation-vigilance measures are markers of ADHD remission. For example, we found this pattern for RTV and target P3 [event-related potential (ERP) of attention allocation] during a reaction-time task under slow-unrewarded (baseline) and fast-rewarded (fast-incentive) conditions (James et al., 2017) (‘Fast task’; Kuntsi et al., 2006). Notably, the ADHD-related impairments in RTV and P3 also showed malleability and improvement under fast-incentive conditions (Cheung et al., 2017). They may thus represent compensatory processes making remitters comparable to controls in their cognitive-neurophysiological profiles.

These findings further our understanding of the cognitive and neural impairments in ADHD persisters and point to initial cognitive-neurophysiological markers of ADHD remission. However, the identified indices represent aggregate measures that may miss systematic and fine-grained aspects of the data due to averaging procedures. Rather than measuring RTV as standard deviation of reaction times (s.d.-RT), sophisticated ex-Gaussian analyses can decompose the reaction times (RTs) and separate extremely slow responses (measured by tau, the exponential component) from the mean (mu) and s.d. (sigma) of the normal RT distribution (Luce, 1991). This approach has consistently shown increased tau in individuals with ADHD compared to controls, while mixed results have been reported for sigma and mu that may reflect subtler impairments (Karalunas, Geurts, Konrad, Bender, & Nigg, 2014; Vainieri et al., 2020). While most studies have focused on children, no study to date has examined ex-Gaussian parameters in adolescents and adults with persistent ADHD. Similarly, finer-grained EEG time-frequency analyses can leverage the millisecond precision of EEG to detect stimulus-related changes in the power and in the variability of the phase (the ‘timing’) of brain oscillations that are not captured by more traditional ERP or quantitative EEG approaches (Loo, Lenartowicz, & Makeig, 2015; Makeig, Debener, Onton, & Delorme, 2004). The few time-frequency studies in ADHD samples to date found lower evoked theta power (reduced attention allocation) (McLoughlin, Palmer, Rijsdijk, & Makeig, 2014; Missonnier et al., 2013), alpha suppression (reduced attentional selection) (Lenartowicz et al., 2014; Ter Huurne et al., 2017), beta suppression (reduced motor preparation) (Hasler et al., 2016; Mazaheri et al., 2014), and more variable theta phase (inconsistency of stimulus processing) (Groom et al., 2010; McLoughlin et al., 2014), compared to controls. During the Fast task, we recently confirmed that adults with ADHD, compared to controls, show lower theta phase consistency, reduced alpha suppression and reduced adjustments between conditions in alpha and beta suppression (Michelini et al., 2018*b*). EEG time-frequency approaches, therefore, hold promise for identifying neural impairments in ADHD, but have not yet been employed to examine the processes underlying ADHD persistence and remission.

In the present study, we aimed to investigate the cognitive and neural processes underlying ADHD remission/persistence using detailed ex-Gaussian and time-frequency EEG measures in a follow-up of adolescents and young adults with and without childhood ADHD. First, given the paucity of previous studies, especially on finer-grained markers of brain oscillations, in adolescents and adults with ADHD, we investigate whether the measures from the baseline and fast-incentive conditions of the Fast task are impaired in ADHD persisters compared to controls (aim 1). Based on previous studies in ADHD samples, including our previous ex-Gaussian and time-frequency analyses using this task in a smaller-scale adult ADHD sample (Michelini et al., 2018*b*; Vainieri et al., 2020), we hypothesize that ADHD persisters are impaired, compared to controls, in measures of attentional fluctuations (tau and sigma), theta power and phase consistency, alpha suppression, and adjustments between conditions in alpha and beta suppression. Second, by examining ADHD remitters, we investigate whether measures that show differences between ADHD persisters and controls are markers of remission at follow-up. We examine ADHD remission with a categorical approach, by comparing remitters to persisters and controls (aim 2a), and with a dimensional approach, by examining the continuous association with ADHD symptoms and functional impairment within participants with childhood ADHD (aim 2b). We hypothesize that all measures showing ADHD persister-control differences also represent markers of remission, consistent with studies using more traditional measures (Cheung et al., 2016; James et al., 2017; Michelini et al., 2016). Third, we hypothesize a significant association between the ex-Gaussian and time-frequency measures that emerged as markers of remission (aim 3), suggestive of common underlying mechanisms.

## Methods

### Sample

The sample used in this study consists of 279 participants, followed-up on average 5.8 years (s.d. = 1.1) after baseline: 110 had a diagnosis of combined-type ADHD based on DSM-IV in childhood (10 sibling pairs and 90 singletons) and 169 were control participants (76 sibling pairs and 17 singletons). Participants with ADHD were recruited from specialized ADHD clinics (Kuntsi et al., 2010) and controls from schools in the UK. Clinical information (neurodevelopmental and psychiatric conditions, and medication use) were collected through neuropsychiatric screening. Exclusion criteria at both assessments included IQ < 70, autism, epilepsy, brain disorders and any medical disorder associated with externalizing behaviours that might mimic ADHD. Other comorbidities were not excluded in order to have an ADHD sample representative of the clinical population. Among participants who took part in the follow-up assessments (*N* = 293), we excluded six controls who met DSM-IV ADHD criteria based on the parent-reported Barkley Informant Rating Scale (Barkley & Murphy, 2006) and six participants with childhood ADHD with missing parent ratings of clinical impairments. Two participants with childhood ADHD, who did not meet ADHD symptom criteria but showed clinical levels of impairment at follow-up, were also excluded to minimize heterogeneity in the sample. Further details on this sample are reported elsewhere (Cheung et al., 2016; Michelini et al., 2018*a*).

Among those with childhood ADHD, 87 (79%) continued to meet clinical (DSM-IV) levels of ADHD symptoms and impairment (ADHD persisters), whereas 23 (21%) were below the clinical cut-off (ADHD remitters). Fourteen ADHD remitters displayed ⩾5 symptoms of inattention or hyperactivity/impulsivity but no functional impairment. Groups were age-matched (mean age = 18.64 across all groups). Of the total, 84% and 82% of participants in the persisters and control groups were males, while 100% of remitted participants were male, as there were no females among ADHD remitters (online Supplementary Table S1). Childhood ADHD participants on medication at follow-up (47%) showed higher ADHD symptoms (*p* < 0.01) and functional impairment (*p* < 0.01) than those not medicated. The proportion of participants on medication did not differ between ADHD persisters and remitters (χ^2^ = 1.95, *p* = 0.16). A 48-h ADHD medication-free period was required prior to assessments. All participants and parents provided informed consent. Study procedures were approved by the London-Surrey Borders Research Ethics Committee (09/H0806/58).

### ADHD diagnosis

The Diagnostic Interview for ADHD in Adults (DIVA) (Kooij et al., 2010) was conducted by trained researchers with parents of ADHD probands to assess DSM-IV-defined ADHD presence/persistence. Raw scores for inattention and hyperactivity/impulsivity symptoms were obtained. Functional impairment was rated from 0 (never or rarely) to 3 (very often) with items from the Barkley's Functional Impairment Scale (Barkley & Murphy, 2006) during interviews with parents. DIVA and functional impairments were used to determine ADHD status, as these were validated against objective markers (cognitive-EEG measures) in this sample, whereas the same objective markers showed limited agreement with self-reported ADHD (Du Rietz et al., 2016). Participants with childhood ADHD were classified as persisters at follow-up if they scored ⩾6 in either the inattention or hyperactivity/impulsivity domains on the DIVA and ⩾2 on at least two areas of impairments; they were classified as remitters otherwise. We defined ADHD outcome using a categorical definition of persistence based on diagnosis and a dimensional approach based on continuous levels of ADHD symptoms and functional impairments.

### IQ

IQ was measured with the Wechsler Abbreviated Scale of Intelligence vocabulary and block design subtests (Wechsler, 1999).

### Task

The task was a computerized four-choice RT task which measures performances under a slow-unrewarded and a fast-incentive condition (Kuntsi et al., 2006). The slow-unrewarded (baseline) condition consists of 72 trials, which followed a standard warned four-choice RT task (online Supplementary Fig. S1). Four empty circles (warning signals, arranged horizontally) first appeared for 8 s, after which one of them (the target) was coloured in. Participants were asked to press the response key that corresponded to the position of the target. Following a response, the stimuli disappeared from the screen and a fixed inter-trial interval of 2.5 s followed. Speed and accuracy were emphasized equally. A comparison condition that used a fast event rate (fore-period of 1 s) and incentives followed immediately after the baseline condition and consisted of 80 trials, with a fixed inter-trial interval of 2.5 s following the response. Participants were told to respond as quickly as possible to win smiley faces and real prizes (£5). The smiley faces appeared below the circles in the middle of the screen when participants responded faster than their own mean RT (MRT) during the baseline condition consecutively for three trials and were updated continuously.

### Ex-Gaussian analysis

We applied ex-Gaussian deconvolution to single-trial RT data employing a maximum-likelihood algorithm, implemented in the QMPE software (Heathcote, Brown, & Cousineau, 2004). This algorithm measures the mean of the normal (Gaussian) component of the RT distribution (mu) and divides the variability into its normal (sigma) and exponential (tau) components. Analyses were performed on participants with >40 RTs from correct responses with plausible RT (>150 ms), as standard procedures in ex-Gaussian analyses (Adamo, Hodsoll, Asherson, Buitelaar, & Kuntsi, 2018; Heathcote, Brown, & Mewhort, 2002).

### EEG recording, pre-processing and analyses

The EEG was recorded from a 62-channel DC-coupled recording system (extended 10–20 montage), using a 500-Hz sampling rate, impedances under 10 kΩ, and FCz as the recording reference. The electro-oculograms were recorded from electrodes above and below the left eye and at the outer canthi. EEG data were pre-processed using Brain Vision Analyzer 2.0 (Brain Products, Gilching, Germany). EEG recordings were down-sampled to 256 Hz, re-referenced to the average of all electrodes (turning FCz into an active channel) and filtered using Butterworth band-pass filters (0.1–30 Hz, 24 dB/octave). Electrical and movement artefacts were removed manually. Trials containing artefacts exceeding ± 100 μV or with a voltage step >50 μV were automatically rejected. Ocular artefacts were corrected using independent component analysis (Jung et al., 2000).

Time-frequency EEG analyses were performed in EEGLAB (Delorme & Makeig, 2004) following procedures adopted in our previous study (Michelini et al., 2018*b*). Modulations of power were quantified with the event-related spectral perturbation (ERSP) index (Delorme & Makeig, 2004). ERSP trials were normalized with respect to the mean log-power spectrum from the pre-stimulus period (−2000 to −1000 ms). Average ERSPs across trials produced a time-frequency representation in decibel (dB) units of increases (red) and decreases (blue) in power with respect to pre-stimulus activity. Phase consistency was calculated with inter-trial phase coherence (ITC), measuring the degree to which the phase of the evoked response is consistent across trials (Makeig et al., 2004). To allow reliable measurement of EEG indices, only participants with ⩾20 artefact-free EEG segments were included in analyses. See online Supplementary material for further details.

**Fig. 1.**
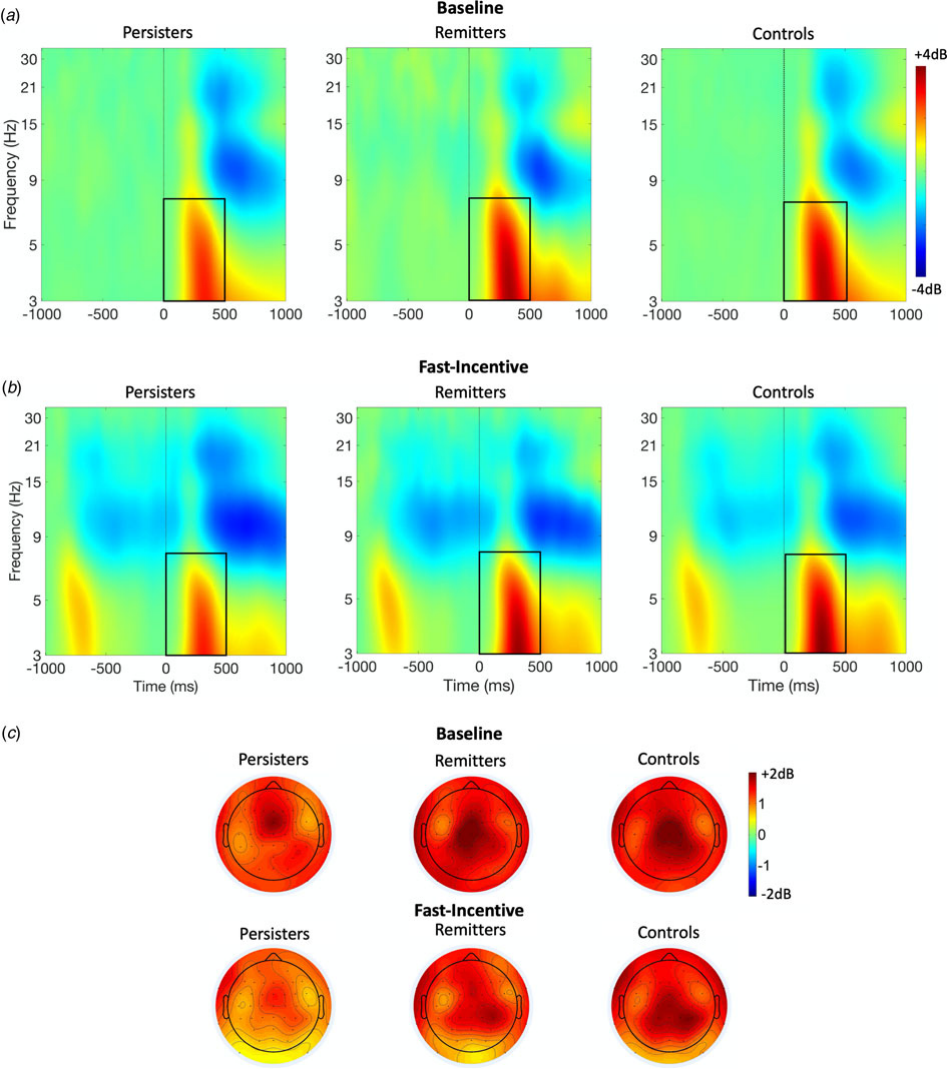
Theta event-related spectral perturbation (ERSP) at centro-parietal regions in ADHD persisters, ADHD remitters and controls across the baseline and fast-incentive conditions of the Fast task. (*a*) ERSP in the baseline condition; (*b*) ERSP in the fast-incentive condition; (*c*) Topographic maps by the group in the 0–500 ms window at each condition.

**Fig. 2.**
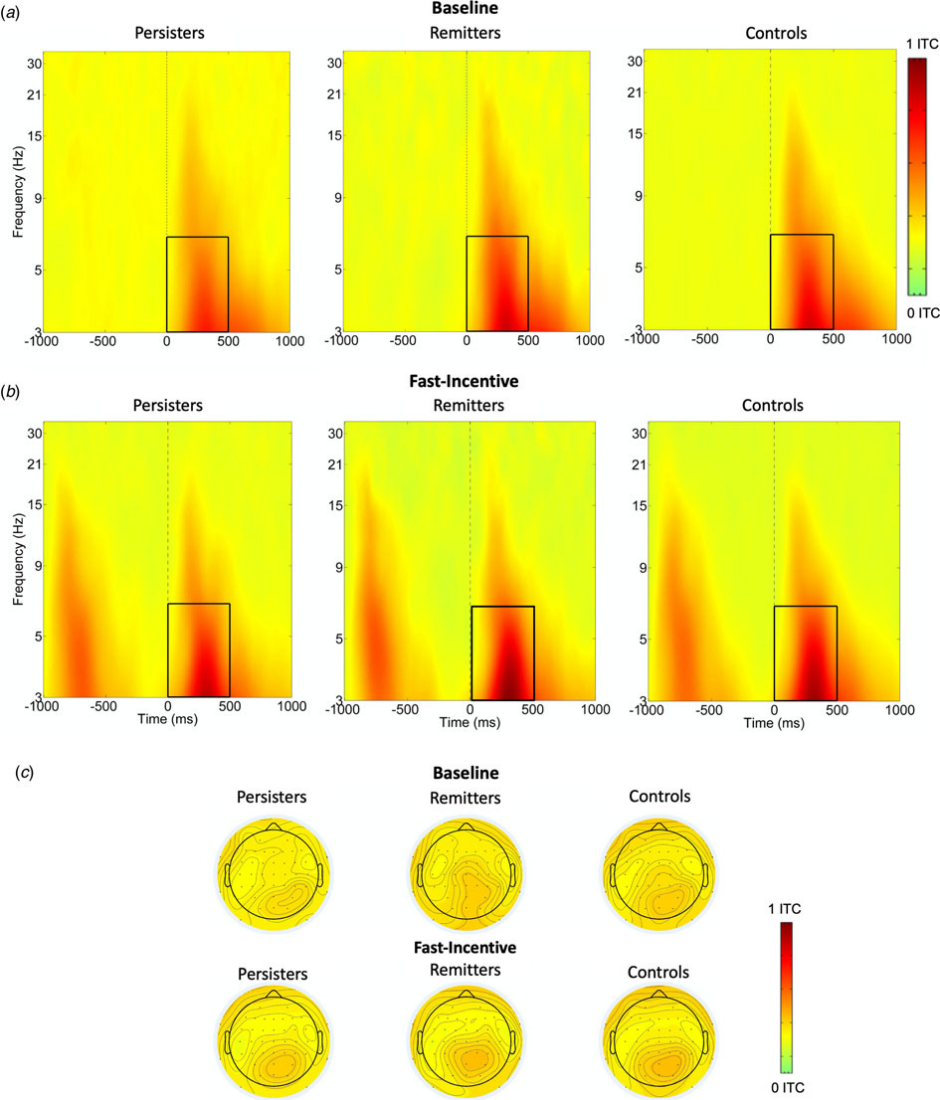
Theta phase consistency at centro-parietal regions in the ADHD persisters, ADHD remitters and controls across the baseline and fast-incentive conditions of the Fast task. (*a*) Theta phase consistency in the baseline condition; (*b*) Theta phase consistency in the fast-incentive condition; (*c*) Topographic maps by the group in the 0–500 ms window at each condition.

ERSP (event-related power) and ITC (phase consistency) were measured in time windows and at scalp locations where they were maximal, following our previous study (Michelini et al., 2018*b*) and other studies on similar attentional processes. Target-related ERSP in theta (3–7 Hz) was measured between 0 and 500 ms over fronto-central regions (average of Fz, F1, F2, FCz, FC1, FC2, Cz, C1, C2) and centro-parietal regions (average of CPz, CP1-CP6, Pz, P3, P4) (DeLosAngeles et al., 2016; Jacobs, Hwang, Curran, & Kahana, 2006), to capture differences in topography across groups and conditions (Fig. 1). Alpha (8–13 Hz) ERSP was measured in two windows (0–500 ms, 500–1000 ms), capturing the broad alpha power modulation, over parieto-occipital regions (average of Pz, P3, P4, P7, P8, POz, PO3, PO4, PO7, PO8) (Bickel, Dias, Epstein, & Javitt, 2012; Mazaheri & Picton, 2005) (online Supplementary Fig. S2). Beta (14–30 Hz) ERSP was extracted between 200 and 700 ms, to measure the shorter target-related beta power suppression over central regions (average of Cz, C1-C4, CPz, CP1-CP4) (Bickel et al., 2012; Mazaheri & Picton, 2005) (online Supplementary Fig. S3). ITC was measured only in theta, given the role of this frequency band in neural consistency (Papenberg, Hämmerer, Müller, Lindenberger, & Li, 2013), between 0 and 500 ms, where greater phase consistency was observed, over centro-parietal regions (average of CPz, CP1-CP6, Pz, P3, P4) (Fig. 2).

### Statistical analyses

For aim 1, we compared ADHD persisters and controls with random intercept linear models (multilevel regression models) investigating main effects of group (ADHD persisters *v*. control), condition (baseline *v.* fast-incentive) and group-by-condition interactions. For measures showing significant (*p* < 0.05) group-by-condition effects, we report pair-wise group comparisons in baseline and fast-incentive conditions separately. For measures showing significant main group effects but non-significant group-by-condition effects, we report pair-wise group comparisons collapsed across conditions. Additional tests followed up significant condition effects to examine within-group changes between conditions, and significant group-by-condition interactions to examine group differences on the change between conditions. Since theta and alpha ERSP indices were measured, respectively, at two scalp regions and two-time windows, we also tested three-way interactions with these additional factors. All models controlled for age and participants at the family level by including random effects to model the non-independence of observations of siblings within families in multilevel random-intercept models (Bauer, Gottfredson, Dean, & Zucker, 2013).

For measures showing ADHD persister-control differences, we ran the same random-intercept models also including ADHD remitters (aim 2a). Because ADHD persisters had a lower IQ than remitters and controls (online Supplementary Table S1), all analyses were rerun controlling for IQ. As groups were not matched on sex, group analyses were further rerun excluding females (15 persisters, 41 control). For between-group comparisons, we report both *p*-values and standardised beta coefficients, which are interpretable as Pearson's correlation coefficients, thus *β* = 0.10 represents a small effect, *β* = 0.30 represents a medium effect and *β* = 0.50 represents a large effect (Cohen, 1988).

We further examined ex-Gaussian and time-frequency measures in relation to ADHD remission with dimensional analyses (aim 2b). Random-intercept linear models were run in all participants with childhood ADHD to investigate the associations of ex-Gaussian and EEG measures significant in aim 1 (dependent variables) with parent-reported ADHD symptoms and functional impairment (independent variables). These models included symptoms-by-condition or impairment-by-condition interactions to test whether associations changed in the two conditions, and three-way interactions as appropriate for measures included in these analyses. Analyses were run clustering for family status and controlling, firstly, for age and sex and, secondly, also for IQ.

Additional random-intercept linear models examined the associations between the ex-Gaussian (dependent variables) and EEG time-frequency measures (independent variables) that emerged as markers of remission from categorical analyses (aim 3). These analyses were run in the full sample and included an interaction between group and EEG measures to investigate if the strength of the associations differed between groups.

In analyses comparing ADHD persisters and controls on all measures (aim 1), we applied multiple testing correction using the false discovery rate (FDR) to reduce type I errors. Analyses for aim 2 and 3 were only run on a restricted set of measures respectively, surviving multiple-testing correction in aim 1 and emerging as markers of remission in aim 2. We, therefore, did not apply further FDR correction and used a nominal significance level (0.05).

Statistical analyses were run in Stata 15 (Stata Corp, College Station, TX). With the exception of beta (that was normally distributed), all other variables showed skewed distributions and were transformed to normal with a logarithmic transformation. Due to technical issues during data collection, RT and EEG data were not available for one ADHD persister and three controls. All participants with RT data had sufficient responses for ex-Gaussian analyses. Six ADHD persisters and five controls were excluded from EEG analyses in the baseline condition, and one control from both conditions, due to having <20 clean EEG segments.

## Results

### Which measures differ between ADHD persisters and controls (aim 1)?

FDR corrections indicated a *p*-value threshold of *p* < 0.043 (see Table 1). A significant group-by-condition interaction emerged for mu, indicating that significant differences between ADHD persisters and controls were significantly greater in the fast-incentive condition than in the baseline condition (Table 1). Sigma, tau, theta ERSP, and theta phase consistency did not show significant group-by-condition effects. Compared to controls, ADHD persisters showed significantly higher sigma and tau, and significantly lower fronto-central and centro-parietal theta ERSP, as well as lower theta phase consistency, in both conditions (Table 1). No significant differences emerged in alpha and beta between ADHD persisters and controls (*p* > 0.1). All RT measures showed within-group decreases from the baseline to the fast-incentive condition (*p* < 0.001), while theta ERSP in both regions and theta phase consistency did not (all *p* > 0.1). Among measures showing the significant within-group change between condition, only mu showed a significant difference between groups in the degree of change between conditions (*p* < 0.001), with persisters changing less than controls (online Supplementary Table S2). Further details on condition and group-by-condition effects are reported in online Supplementary material.

**Table 1.** Group comparisons on ex-Gaussian and EEG time-frequency measures in the baseline and fast-incentive conditions and across conditions

	Baseline condition						Fast-incentive condition					
	Aim 1		Aim 2a				Aim 1		Aim 2a			
	ADHD persisters *v*. controls		ADHD persisters *v*. remitters		ADHD remitters *v*. controls		ADHD persisters *v*. controls		ADHD persisters *v*. remitters		ADHD remitters *v*. controls	
	*β* (95% CI)	*p*	*β* (95% CI)	*p*	*β* (95% CI)	*p*	*β* (95% CI)	*p*	*β* (95% CI)	*p*	*β* (95% CI)	*p*
**Mu**	0.22 (0.01 to 0.44)	0.043*	0.18 (−0.19 to 0.56)	0.332	0.04 (−0.32 to 0.40)	0.823	**0.50 (0.27 to 0.71)**	<0.001**	*0.40* (*0.02 to 0.77*)	0.037*	0.10 (−0.26 to 0.46)	0.583

	Across condition					
	Aim 1		Aim 2a			
	ADHD persisters *v*. controls		ADHD persisters *v*. remitters		ADHD remitters *v*. controls	
	*β* (95% CI)	*p*	*β* (95% CI)	*p*	*β* (95% CI)	*p*
**Sigma**	*0.33* (*0.15 to 0.53*)	<0.001**	*0.31* (*−0.08 to 0.65*)	0.064	0.01 (−0.30 to 0.34)	0.916
**Tau**	**0.74 (0.56 to 0.93)**	<0.001**	*0.42* (*0.16 to 0.81*)	0.003*	0.26 (−0.05 to 0.57)	0.101
**Theta ERSP FC**	−0.20 (−0.44 to −0.11)	0.003*	−0.24 (−0.51 to 0.03)	0.081	−0.03 (−0.30 to 0.22)	0.784
**Theta ERSP CP**	**−0.53 (−0.66 to −0.32)**	<0.001**	*−0.44* (*−0.82 to −0.08*)	0.015*	−0.08 (−0.44 to 0.27)	0.627
**Theta phase consistency**	*−0.43* (*−0.67 to −0.20*)	<0.001**	*−0.42* (*−0.79 to −0.04*)	0.027*	−0.01 (−0.38 to 0.34)	0.939

ADHD, attention-deficit/hyperactivity disorder; ERSP, event-related spectral perturbation; FC, fronto-central; CP, centro-parietal.

*Notes*: For aim 1, the *p*-value threshold surviving multiple testing correction was determined as 0.043 using false discovery rate (FDR). Post-hoc tests are reported by condition only for measures showing significant group-by-condition effects. For measures showing non-significant group-by-condition effects, post-hoc tests are reported across conditions. Ex-Gaussian variables were available for 86 persisters, 23 remitters and 166 controls. ERSP and theta phase consistency variables were available for 81 persisters, 23 remitters and 163 controls. **p < 0.01, *p < 0.05. Bold = large effect size (*β* ⩾ 0.50); Italics = medium effects size (*β* ⩾ 0.30).

ADHD persister-control differences in mu became non-significant in both conditions when controlling for IQ (online Supplementary Table S3) and in the baseline condition in the male-only sample (online Supplementary Table S4).

### Which measures are markers of remission (aim 2a and 2b)?

Analyses were restricted to measures that survived multiple testing corrections in the analysis of aim 1. In categorical analyses (aim 2a) on ADHD remitters, persisters and controls, remitters did not significantly differ from controls on any other measure (Table 1). Mu, which showed a significant group-by-condition interaction, was lower in ADHD remitters compared to persisters in the fast-incentive condition, but no differences emerged in the baseline condition (Table 1). ADHD remitters further showed lower tau, as well as greater centro-parietal theta ERSP and theta phase consistency compared to persisters (Table 1, Figs 1 and 2). ADHD remitters showed significant within-group changes between conditions in all ex-Gaussian measures (all *p* < 0.05) but not in theta ERSP and phase consistency measures (all *p* > 0.1) Full details on condition and group-by-condition effects are reported in online Supplementary material.

The ADHD remitter-persister differences in mu in the fast-incentive condition became non-significant when controlling for IQ (online Supplementary Table S3) and in the male-only sample (online Supplementary Table S4).

Dimensional analyses (aim 2b) in participants with childhood ADHD, controlling for sex and age, showed non-significant associations of ADHD symptoms with all ex-Gaussian and time-frequency measures (Table 2). These associations did not differ between conditions, as indicated by non-significant interactions between ADHD symptoms and condition for all measures (all *p* > 0.1). Mu showed a significant interaction between functional impairment and condition (*p* = 0.024): functional impairment was associated with mu in the fast-incentive condition but not in the baseline condition (Table 2). Functional impairment was significantly associated with tau irrespective of condition (Table 2), as the functional impairment-by-condition interaction was non-significant. The other measures were not associated with functional impairment and the functional impairment-by-condition interactions were non-significant (all *p* < 0.1). When also controlling for IQ, the association of functional impairment with mu and tau became non-significant (online Supplementary Table S5).

**Table 2.** Random-intercept linear models of ex-Gaussian and EEG time-frequency measures with parent-reported ADHD symptoms and impairment within the ADHD group only, controlling for age and sex

		ADHD symptoms		Functional impairment	
Aim 2b		*β* (95% CI)	*p*	*β* (95% CI)	*p*
**Mu**		<0.00 (−0.31 to 0.30)	0.983	–	–
	Baseline	–	–	−0.03 (−0.21 to 0.14)	0.701
	Fast-incentive	–	–	0.20 (0.01 to 0.39)	0.033*
**Sigma**		0.13 (−0.34 to 0.60)	0.580	−0.06 (−0.58 to 0.46)	0.827
**Tau**		0.27 (−0.05 to 0.59)	0.095	*0.37* (*0.01 to 0.72*)	0.043*
**Theta ERSP CP**		0.03 (−0.13 to 0.90)	0.147	−0.04 (−0.60 to 0.30)	0.523
**Theta phase consistency**		0.03 (−0.34 to 0.39)	0.888	−0.13 (−0.53 to 0.26)	0.513

ADHD, attention-deficit/hyperactivity disorder; ERSP, event-related spectral perturbation; CP, centro-parietal.

*Notes*: Ex-Gaussian variables were available for 87 persisters, 23 remitters, and 169 controls. ERSP and theta phase consistency variables were available for 81 persisters, 23 remitters, and 163 controls. **p < 0.010, *p < 0.050. Bold = large effect size (*β* ⩾ 0.50); Italics = medium effects size (*β* ⩾ 0.30). Analyses of ADHD symptoms and impairment with all variables, as well as for mu with ADHD symptoms, were run collapsing across baseline and fast-incentive conditions, as the interactions with the condition were non-significant (*p* > 0.10).

### Are ex-Gaussian and EEG time-frequency markers of remission associated with each other (aim 3)?

We examined the association of mu in the baseline and fast-incentive condition separately and tau across conditions with centro-parietal theta ERSP and phase consistency, as these measures emerged as markers of remission in categorical analyses. Mu showed a significant negative association with theta ERSP and theta phase consistency in both conditions (online Supplementary Table S6), while the interactions between group and theta ERSP or theta phase consistency were non-significant, indicating that the groups did not differ on the strength of these associations. Similarly, tau across conditions showed a significant negative association with theta ERSP and phase consistency, while the interactions with the group were non-significant (online Supplementary Table S6).

## Discussion

In a first large-scale investigation to examine ex-Gaussian and EEG time-frequency markers in adolescents and adults with childhood ADHD, we observed widespread impairments in ADHD persisters, compared to controls, in ex-Gaussian measures of response variability (sigma and tau) and response speed (mu), and in neurophysiological markers of neural variability (theta phase consistency) and attention allocation (theta ERSP). We further identified several potential new markers of remission, on which ADHD remitters were comparable to controls but significantly different from persisters: mu, tau, centro-parietal theta ERSP and theta phase consistency. The ex-Gaussian and EEG markers of remission were significantly associated with each other, indicating they may reflect partly overlapping processes. The measures emerging as potential markers of remission represent possible compensatory mechanisms in ADHD remitters, extending our previous findings on more traditional cognitive-performance and ERP measures (Cheung et al., 2016; James et al., 2017; Michelini et al., 2016).

ADHD persisters showed increased cognitive variability compared to controls (with large effect sizes), consistent with our hypotheses and previous ex-Gaussian studies in individuals with ADHD (Buzy, Medoff, & Schweitzer, 2009; Vainieri et al., 2020; Vaurio, Simmonds, & Mostofsky, 2009). We also observed increased mu in ADHD persisters compared to controls, despite some previous studies not detecting this potentially subtler impairment (Gmehlin et al., 2014; Lin, Hwang-Gu, & Gau, 2015). In this largest time-frequency analysis of ADHD to date, we further report that individuals with persistent ADHD, compared to controls, show lower theta phase consistency and evoked theta power, reflecting lower consistency of neural stimulus processing across trials (Makeig et al., 2004) and lower attentional processing (Klimesch, Sauseng, & Hanslmayr, 2007), respectively, confirming previous evidence in smaller ADHD samples (Groom et al., 2010; McLoughlin et al., 2014; Michelini et al., 2018*b*; Missonnier et al., 2013). We did not find differences between ADHD persisters and controls on alpha suppression, nor on adjustments between conditions in alpha and beta, contrary to our predictions based on the ADHD-control differences in our previous smaller-scale time-frequency study (Michelini et al., 2018*b*). Such inconsistencies may be explained by sex differences (the current study primarily included males, while the previous one only females) or age (the current sample was younger). These findings advance our understanding of the cognitive and neural correlates of persistent ADHD in adolescence and early adulthood, showing specific RT and brain-oscillatory impairments in measures mapping onto attention-vigilance processes.

We further examined ex-Gaussian and brain-oscillatory measures in relation to ADHD remission, both categorically and dimensionally. Results for mu showed that ADHD remitters were comparable to controls and significantly different from persisters in the fast-incentive condition, but did not differ significantly from either controls or persisters in the baseline condition. ADHD remitters were also comparable to controls but different from persisters on tau across conditions. These findings suggest that tau may be considered a marker of ADHD remission in both conditions, while mu may be sensitive to remission only in the fast-incentive condition. This pattern potentially indicates residual impairments in mu in the remitted group in the baseline condition, which is more challenging for ADHD participants due to the long inter-trial interval. Conversely, the significantly lower mu in remitters than in persisters in the fast-incentive condition may suggest that compensatory processes might arise in a more engaging context. Results of dimensional analyses were consistent with these categorical findings, as tau across conditions and mu in the fast-incentive condition were continuously associated with functional impairment in individuals with childhood ADHD. For sigma, we observed no differences between remitters and the other groups or continuous associations with ADHD symptoms or functional impairments, indicating that this measure may not be a marker of remission. At the neural (EEG) level, ADHD remitters were comparable to controls but showed significantly higher centro-parietal theta power and theta phase consistency compared to persisters, suggesting that these variables are potential markers of remission. Yet, they were not dimensionally associated with ADHD symptoms or functional impairment, suggesting that the pattern of remission for these variables should be investigated further in future research. In further analyses controlling for IQ, results for tau and centro-parietal theta power were unchanged, indicating they are markers of remission independently of IQ, while results for other measures became non-significant. Taken together, the current results provide novel evidence that markers of attention-vigilance processes, including ex-Gaussian measures of response speed (mu), variability of long responses (tau) and EEG power and phase consistency in theta oscillations, may be implicated in ADHD remission, consistent with previous findings on RTV measured as s.d.-RT and P3 during this task (James et al., 2017).

In examining the association between the identified ex-Gaussian and EEG markers of remission, we found a significant association of evoked theta power and theta phase consistency with mu and tau. These results indicate that alterations in theta oscillations may partly underlie atypical response speed and variability of long responses. Future studies should replicate these associations and further investigate their possible underlying etiological processes. Of note, while all groups showed significant improvements in ex-Gaussian measures from the baseline to the fast-incentive condition, in line with previous findings on RTV (Cheung et al., 2016), no improvement emerged in theta power and phase variability. As such, these brain markers of remission may be less malleable than cognitive markers of remission.

The following limitations should be considered. First, the high ADHD persistence rate at follow-up resulted in a small group of remitters; thus, some non-significant differences between ADHD remitters and the other groups might be due to low power. Although we successfully detected medium-to-large effect sizes in markers of remission with current sample sizes and also ran dimensional analyses, future studies should include a larger remitted group. Second, groups were not matched on sex and the small number of females did not allow us to directly examine sex differences. Yet, results in the male-only sample showed comparable effect sizes to those in the full sample, indicating that reduced significance for some effects after excluding females may thus have arisen from the smaller size in the male-only sample. Third, since participants were adolescents and young adults, who may still be undergoing cortical maturation and could potentially remit at an older age, further follow-ups are required to confirm the applicability of these findings to older individuals. Fourth, although this study was conducted on the adolescent and young adult follow-up assessments of a sample of children with ADHD and controls, different cognitive-EEG batteries at the childhood and follow-up assessments precluded us from conducting formal longitudinal analyses. Our previous study on the childhood data showed no childhood differences between participants whose ADHD persisted and remitted at follow-up on cognitive measures related to those emerging here as markers of remission (e.g. RTV measured as RT-s.d.) (Cheung et al., 2015). This might suggest that the differences at follow-up reported here between remitters and persisters were likely not explained by pre-existing differences in childhood. Nevertheless, since this is a common limitation among studies of ADHD remission and persistence (Franke et al., 2018), future studies using repeated cognitive and brain measures across development are warranted.

In conclusion, our cognitive-EEG investigation shows that detailed measures of response speed emerge as potential markers of ADHD remission, under more engaging (fast-incentive) conditions, while measures of neural markers of phase variability (i.e. lower theta phase consistency) and attention allocation (cento parietal theta power), as well as attentional lapses (tau), emerged as markers of remission independently of the condition. These measures may point to potential compensatory mechanisms linked to remission of ADHD from childhood to adulthood, extending our previous findings on more traditional measures of attention-vigilance processes (Cheung et al., 2016; James et al., 2017; Michelini et al., 2016).

## Supplementary material

For supplementary material accompanying this paper visit https://doi.org/10.1017/S0033291720002056. click here to view supplementary material
