# Supplementary material for: Event-related brain-oscillatory and ex-Gaussian markers of remission and persistence of ADHD
Source: Psychol Med. 2020 Jul 2;52(2):352–61. doi: 10.1017/S0033291720002056 (PMC8842193; doi:10.1017/S0033291720002056)
Supplement: Supplementary file 1 [file S0033291720002056sup001.docx]

**Supplementary material**

***Event-related brain oscillatory and ex-Gaussian markers of remission and persistence of ADHD***

*Isabella Vainieri, Giorgia Michelini, Nicoletta Adamo, Celeste H. M. Cheung, Philip Asherson, Jonna Kuntsi*

**Further details on the event-related perturbation (ERSP) analysis**

Time-frequency analyses were conducted using the ERSP index calculated in the EEGLAB toolbox. Specifically, ERSP values were computed from -2 to 2 s centred around target onset with a Morlet wavelet decomposition of frequencies between 3 and 30 Hz, with linearly increasing number of cycles (frequency step of 0.80 Hz) from 2 cycles for the lowest frequency (3 Hz) to 24.60 cycles for the highest frequency (30 Hz). The modulations of EEG frequency components in response to a stimulus are normalized with respect to spectral power in a pre-defined pre-stimulus period. Specifically, the post-stimulus power at each time-frequency point is divided by the mean spectral power in the pre-stimulus period (typically reflecting spontaneous EEG) at the same frequency. The normalized post-stimulus signal is scaled in decibel (dB), a logarithmic unit that represents the ratio of two signals. When comparing the ERSPs in two conditions, it is necessary to match the pre-stimulus period used to normalize the post-stimulus ERSPs (Herrmann *et al*. 2014). Phase consistency was calculated with inter-trial phase coherence (ITC), measuring the degree to which the phase of the evoked response (derived from the Morlet wavelet used for ERSPs) is consistent across trials (Tallon-Baudry *et al.* 1996; Delorme & Makeig 2004; Makeig *et al.* 2004). ITC is independent of power and ranges from 0 (no phase consistency) to 1 (perfect phase consistency).

Consistent with our previous time-frequency publication using this task (Michelini *et al.* 2018b), to compare the ERSPs in the baseline and fast-incentive conditions, we matched the timing of the pre-stimulus window across the two conditions (-2 to -1 s) with respect to the appearance of the target stimulus (Figure S1). In the fast-incentive condition, this window represents the 1 s period during a 2.5-s fixed inter-trial interval between the end of a trial and the subsequent warning, appearing 1 s before the target. During the -2 to -1 s period in this condition, participants are inactive (i.e., waiting for the target) while viewing a “+” fixation and as many smiley faces as they have accumulated over the course of the fast-incentive condition. The same corresponding -2 to -1 s window was used as the pre-stimulus period in the baseline condition, which similarly represents an inactive period during the long fore-period (8 s) between the appearance of the warning and of the target. This window in the baseline condition was chosen instead of a pre-warning window in the baseline condition (-9 to -8 s) in order to use pre-stimulus periods with identical time lags before the target onset (-2 to -1 s) in both conditions.

**Further details on categorical analyses**

*Which measures differentiate between ADHD persisters and controls (aim 1)?*

Significant group, condition and group-by-condition interaction (all p<0.001) effects emerged for mu. ADHD persisters showed greater mu than controls in both conditions (Table 1). Mu was greater in both groups in the baseline conditions compared to the fast-incentive condition (p<0.001), but the degree of change was greater in controls compared to ADHD persisters. Significant group and condition effects (both p<0.001), but non-significant group-by-condition interactions (both p>0.10), emerged for sigma and tau. Sigma and tau were significantly increased in ADHD persisters compared to controls across conditions (Table 1). Both measures were higher in the baseline condition compared to the fast-incentive condition.

Theta ERSP showed a significant effect of group (p<0.001) and region (p<0.001), but no effect of condition (p=0.347) or group-by-condition-by-region interaction (p=0.298). After removing the three-way interaction, theta in both regions showed a significant group effect (p<0.001), but no significant condition effect (p=0.229 at fronto-central site and p=0.745 at centro-parietal site) or group-by-condition interaction (p=0.698 and p=0.469, respectively). Theta ERSP across regions was significantly decreased in ADHD persisters compared to controls (Table 1).

Alpha ERSP showed a significant condition effect (p<0.001), but no group effects (p=0.799) or group-by-condition-by-time interaction (p=0.511). After removing the three-way interaction, in both time windows there was a significant condition effect (p<0.001), but no group effect (p=0.585 and p=0.853 respectively) or group-by-condition interaction (p=0.856 and p=0.395 respectively), indicating no differences between ADHD persisters and controls on this measure. In both groups alpha suppression was greater in the fast-incentive condition compared to the baseline condition.

Beta ERSP showed a significant main effect of condition (p<0.001), but no significant main effect of group (p=0.051) and group-by-condition interaction (p=0.968), indicating no differences between ADHD persisters and controls on this measure. In both groups, beta suppression was greater in the fast-incentive condition compared to the baseline condition.

A significant group effect (p<0.001), but no condition effect (p=0.455) or group-by-condition interaction (p=0.758), emerged for theta phase consistency. Theta phase consistency was significantly lower (i.e., greater phase variability) in ADHD persisters than controls across task conditions (Table 1, Figure 2).

*Which measures are markers of remission (aim 2a and 2b)?*

Mu showed significant group (p<0.001), condition (p<0.001) and group-by-condition interaction (p=0.032) effects. Sigma and tau showed significant group and condition effects (both p<0.001), but non-significant group-by-condition interactions (both p>0.10). Pair-wise comparisons for mu in the baseline condition and sigma across conditions between ADHD remitters and persisters, as well as between ADHD remitters and controls, were not significant (Table 1). Tau across conditions showed a significant difference between ADHD remitters and persisters and a non-significant difference between ADHD remitters and controls (Table 1). In the fast-incentive condition, ADHD remitters showed significantly lower mu than persisters. Remitters did not differ from controls on any ex-Gaussian measure (Table 1). ADHD remitters showed a significant decrease in all ex-Gaussian measures from the baseline to the fast-incentive condition (all p<0.001). For mu, the degree of change between conditions in ADHD remitters did not differ from either persisters or controls (Table S2).

Theta ERSP showed a significant effect of group (p<0.001) and region (p=0.003), but no effect of condition (p=0.675) or group-by-condition-by-region interaction (p=0.485). After removing the three-way interaction, theta in both regions showed a significant group effect (p<0.001), but no significant condition effect (p=0.331 and p=0.798, respectively) or group-by-condition interaction (p=0.916 and p=0.601, respectively). ADHD remitters did not differ on centro-parietal theta from controls, but showed a significant difference from persisters in the post hoc analyses across conditions (Table 1, Figure 1), while remitters did not differ from controls or persisters on fronto-central theta (Table 1, Figure 1).

A significant group effect (p<0.001), but no condition effect (p=0.328) or group-by-condition interaction (p=0.398), emerged for theta phase consistency. ADHD remitters showed significantly higher theta phase consistency than persisters but did not differ from controls when performing post hoc analyses across conditons (Table 1, Figure 2).

**Results covarying for IQ**

ADHD persisters in this sample had a lower IQ than ADHD remitters and controls, and childhood IQ predicted ADHD outcome at follow up (Cheung *et al.* 2015). To examined whether group differences on IQ contributed to the results on ex-Gaussian and time-frequency analyses, we re-run all analyses controlling for IQ (Table S2-S3).

*Which measures differentiate between ADHD persisters and controls covarying for IQ (aim 1)?*

Controlling for IQ, effects of condition and group-by-condition interaction on mu, sigma and tau were unchanged (p<0.001 and p=0.009 respectively for mu; p<0.001 and p=0.629 respectively for sigma; p<0.001 and p=0.475 respectively for tau), but main group effects became non-significant for mu and sigma (p=0.392 and p=0.173, respectively). Group differences on tau between ADHD persisters and controls remained unchanged across conditions (p<0.10). In both groups, the within-group change between conditions for all measures, as well as the degree of change between conditions in mu, remained unchanged (all p<0.001) (Table S2).

The main effects of group, condition and group-by-condition interaction did not change in theta ERSP at fronto-central regions (p=0.001, p=0.202 and p=0.646) or centro-parietal regions (p<0.001, p=0.759 and p=0.454). Group differences between ADHD persisters and controls remained unchanged across conditions for theta ERSP in both regions (Table S3).

The main effects of group, condition and group-by-condition interaction at both time windows did not change for alpha ERSP (p=0.518, p<0.001, p=0.852; and p=0.867, p<0.001, p=0.392 respectively). In both groups, the within-group change between condition also did not change in either time window (all p<0.001).

For beta ERSP, the main effect of group, condition and group-by-condition interaction remained unchanged (p=0.057, p<0.001 and p=0.829 respectively). Within-group change between condition also remained unchanged in both groups (all p<0.001).

Main effects of group, condition, and group-by-condition interaction did not change in theta phase consistency when covarying for IQ (p=0.003, p=0.463, and p=0.735 respectively). Group differences in theta phase consistency between ADHD persisters and controls remained unchanged across conditions (Table S3).

*Which measures are markers of remission (aim 2a and 2b)?*

In categorical analyses (aim 2a), controlling for IQ, effects of condition and group-by-condition interaction on mu and sigma were unchanged (p<0.001 and p=0.032, respectively, for mu; p<0.001 and p=0.852, respectively, for sigma), but the main group effect for mu and sigma became non-significant (p=0.293 and p=0.512, respectively). For tau, the group, condition and group-by-condition interaction effects were unchanged (p<0.001, p<0.001 and p=0.416, respectively). Group differences in mu between ADHD persisters and remitters became non-significant in the fast-incentive condition (table S3). Since the effect of group and group-by condition interaction was not significant for sigma, pair-wise group comparisons in each condition separately were not run for this variable (Table S3). For tau, group differences between ADHD persisters and remitters, and between ADHD remitters and controls remained unchanged (Table S3). The within-group change between conditions in ADHD remitters for all measures (all p<0.001), as well as the degree of change between conditions in ADHD remitters compared to persisters and controls, remained unchanged (Table S2).

The main effects of group, condition and group-by-condition interaction did not change in theta ERSP at fronto-central regions when controlling for IQ (p<0.002, p=0.672 and p=0.497). Group differences between ADHD persisters and remitters in theta at the centro-parietal regions remained unchanged (Table S3).

Main effects of group, condition, and group-by-condition interaction did not change in theta phase consistency when covarying for IQ (p=0.047, p=0.379 and p=0.615, respectively). Group differences on theta phase consistency ADHD persisters and remitters became non-significant across conditions (Table S3).

In dimensional analyses (aim 2b), when controlling for IQ, results remained mostly unchanged except for the post-hoc test for mu in the fast-incentive condition and the association between tau and impairment (both of which became non-significant; Table S5).

**Categorical analyses in the male-only sample**

The majority of individuals in our sample (80%) were males. Since groups were not fully matched on sex, analyses were repeated with females (15 ADHD persisters, 41 controls) removed (Table S4).

*Which measures differentiate between ADHD persisters and controls in the male-only sample (aim 1)?*

Effects of group, condition and group-by-condition interaction on mu, sigma and tau were unchanged (p=0.003, p<0.001 and p=0.04 for mu; p<0.001, p<0.001 and p=0.636 for sigma; p<0.001, p<0.001 and p=0.476 for tau). Group differences on mu between ADHD persisters and controls became non-significant in the baseline condition, while differences on sigma and tau between ADHD persisters and controls remained unchanged across conditions (Table S4). The within-group change between conditions for both groups, as well as the degree of change between conditions in ADHD persisters compared to controls in mu, remained unchanged (Table S2).

The main effects of group, condition and group-by-condition interaction did not change for theta ERSP at fronto-central regions (p=0.001, p=0.229 and p=0.697) or centro-parietal regions (p<0.001, p=0.745 and p=0.469). Group differences between ADHD persisters and controls remained unchanged across conditions (Table S4).

The main effects of group, condition and group-by-condition interaction at both time windows did not change for alpha ERSP (p=0.956, p<0.001, p=0.393; and p=0.570, p<0.001, p=0.676, respectively). The within-group change between condition in both groups also did not change in either time window (all p<0.001).

For beta ERSP, the main effect of group, condition and group-by-condition interaction remained unchanged (p=0.469, p<0.001 and p=0.859, respectively). The within-group change between condition remained unchanged for both groups (both p<0.001).

Main effects of group, condition and group-by-condition interaction did not change for theta phase consistency in the male-only sample (p=0.002, p=0.956 and p=0.740, respectively). Group differences on theta phase consistency between ADHD persisters and controls remained unchanged (Table S3).

*Which measures are markers of remission in the male-only sample?*

Group and condition effects did not change for mu, sigma or tau when repeating the analyses in the male-only sample (p<0.001, p<0.001 and p>0.10, respectively, for all measures), while group-by-condition interaction effect became non-significant for mu only (p=0.115). The significant differences in mu between ADHD persisters and remitters became non-significant in the fast-incentive condition (Table S4). The significant differences in tau between ADHD persisters and remitters remain unchanged. The within-group change between conditions remained unchanged in ADHD remitters for all measures (all p<0.001).

Main effects of group, condition and group-by-condition interaction did not change for theta ERSP in either region when repeating the analyses in the male-only sample (p<0.05, p>0.100 and p>0.100). The differences between persisters and remitters in theta ERSP for both regions remained unchanged across conditions (Table S4).

Main effects of group, condition and group-by-condition interaction did not change in theta phase consistency in the male-only sample (p=0.004, p=0.373, and p=0.487 respectively). Group differences on theta phase consistency between ADHD remitters and persisters, and between ADHD remitters and controls remained unchanged across conditions (Table S4).

**Table S1.** Sample demographics divided by group, with test for group difference

|  | **ADHD persisters** | **ADHD remitters** | **Controls** | **Group comparison** | | | |
| --- | --- | --- | --- | --- | --- | --- | --- |
| **Gender** | **%** | **%** | **%** | **p** | **ADHD persisters vs controls** | **ADHD persisters vs remitters** | **ADHD remitters vs controls** |
|  |  |  |  |  | **p** | **p** | **p** |
| **Male** | 84% | 100% | 81% | 0.02* | 0.24 | 0.03* | <0.01** |
| **Age** | 18.27 (3.03) | 18.89 (3.06) | 18.77 (2.19) | 0.15 | **-** | **-** | **-** |
| **IQ** | 96.20 (15.33) | 104.57 (13.63) | 109.98 (12.42) | <0.01** | <0.01** | 0.02* | 0.10 |
| **ADHD symptoms** | 14.13  (2.82) | 9.17  (4.16) | NA | NA | NA | <0.01** | NA |
| **Functional impairment** | 16.44  (5.31) | 5.56  (3.64) | NA | NA | NA | <0.01** | NA |

Abbreviations: ADHD, attention-deficit/hyperactivity disorder. Notes: F, females, M, males. Group differences on gender were tested via Chi-square test; group differences on age and IQ were tested with regression models. Group differences in gender, age and IQ were reported in a previous paper on this sample (Cheung et al. 2016; Michelini et al. 2016). Since diagnostic interviews were not conducted in controls, descriptive statistics and group comparisons are provided for the ADHD persisters and remitters only. **p<0.01; *p<0.05.

**Table S2** Within-group analyses and between-group comparisons of condition effects for mu

| **Measures** | **Within-group differences from the baseline to the fast-incentive condition** | | | **Between-group comparisons of changes between conditions** | | | | | |
| --- | --- | --- | --- | --- | --- | --- | --- | --- | --- |
|  | **ADHD persisters** | **ADHD remitters** | **Control** | **Aim 1** | | **Aim 2a** | | | |
|  |  |  |  | **ADHD persisters vs controls** | | **ADHD persisters vs remitters** | | **ADHD remitters vs controls** | |
|  | ***p*** | ***p*** | ***p*** | ***β* *(95% CI)*** | ***p*** | ***β* *(95% CI)*** | ***p*** | ***β (95% CI)*** | ***p*** |
| Mu | p<0.001** | p<0.001** | p<0.01** | -0.49 (-0.48; -0.14) | 0.009* | 0.21 (-0.14; 0.57) | 0.249 | 0.05 (-0.28; 0.40) | 0.733 |
| Mu covarying for IQ | p<0.01** | p<0.01** | p<0.01** | -0.48 (-0.49; -0.07) | 0.009* | 0.21 (-0.14; 0.57) | 0.249 | 0.06 (-0.28; 0.40) | 0.733 |
| Mu in the male-only sample | p<0.01** | p<0.01** | p<0.01** | -0.32 (-0.54; -0.11) | 0.003* | - | - | - | - |

Abbreviations: ADHD, attention-deficit/hyperactivity disorder. Notes: **p<0.01, *p<0.05. Bold=large effect size (β≥.50); Italics=medium effects size (β≥.30).

**Table S3.** Group comparisons on ex-Gaussian and EEG time-frequency measures in the baseline and fast-incentive conditions and across conditions covarying IQ

|  | **Baseline condition** | | | | | | **Fast-incentive condition** | | | | | | |
| --- | --- | --- | --- | --- | --- | --- | --- | --- | --- | --- | --- | --- | --- |
|  | **Aim 1** | | **Aim 2a** | | | | **Aim 1** | | **Aim 2a** | | | | |
|  | **ADHD persisters vs controls** | | **ADHD persisters vs remitters** | | **ADHD remitters vs controls** | | **ADHD persisters vs controls** | | **ADHD persisters vs remitters** | | **ADHD remitters vs controls** | | |
|  | ***β (95% CI)*** | ***p*** | ***β (95% CI)*** | ***p*** | ***β (95% CI)*** | ***p*** | ***β (95% CI)*** | ***p*** | ***β (95% CI)*** | ***p*** | ***β (95% CI)*** | ***p*** |  |
| **Mu** | 0.05 (-0.27; 0.18) | 0.685 | 0.05 (-0.30; 0.41) | 0.772 | -0.09 (-0.44; 0.24) | 0.582 | 0.22 (0.03; 0.45) | 0.053 | 0.26 (-0.09; 0.62) | 0.145 | -0.03 (-0.38; 0.30) | 0.833 |  |

**Collapsed across condition**

|  | | **Aim 1** | | **Aim 2a** | | | |
| --- | --- | --- | --- | --- | --- | --- | --- |
|  |  | **ADHD persisters vs controls** | | **ADHD persisters vs remitters** | | **ADHD remitters vs controls** | |
|  |  | ***β (95% CI)*** | ***p*** | ***β (95% CI)*** | ***p*** | ***β (95% CI)*** | ***p*** |
| **Tau** | | **0.57 (0.35; 0.773)** | <0.001** | *0.40 (0.07; 0.71)* | 0.014* | 0.17 (-0.13; 0.47) | 0.263 |
| **Theta ERSP** | FC | -0.23 (-0.42; -0.04) | 0.013* | -0.22 (-0.49; 0.05) | 0.117 | -0.01 (-0.28; 0.25) | 0.918 |
|  | CP | *-040 (-0.65; -0.15)* | <0.001** | *-0.38 (-0.74; -0.02)* | 0.037* | -0.02 (-0.37; 0.33) | 0.901 |
| **Theta ITC** | | *-0.34 (-0.54; -0.03)* | 0.026* | -0.28 (-0.72, 0.26) | 0.069 | -0.05 (-0.30; 0.42) | 0.748 |

Abbreviations: ADHD, attention-deficit/hyperactivity disorder; ERSP, event-related spectral perturbation; FC, fronto-central; CP, centro-parietal. Notes: For aim 1, the p-value threshold surviving multiple testing correction was determined as 0.026 using false discovery rates (FDR). Post-hoc tests are reported by condition only for measures showing significant group-by-condition effects. For measures showing non-significant group-by-condition effects, post-hoc tests are reported across conditions. Ex-Gaussian variables were available for 86 persisters, 23 remitters, and 166 controls. ERSP and theta phase consistency variables were available for 81 persisters, 23 remitters, and 163 controls. **p<0.01, *p<0.05. Bold=large effect size (β ≥.50); Italics=medium effects size (β≥

.30).

**Table S4.** Group comparisons on ex-Gaussian and EEG time-frequency measures in the baseline and fast-incentive conditions and across conditions in the male participants only

|  | **Baseline condition** | | | | | | **Fast-incentive condition** | | | | | | |
| --- | --- | --- | --- | --- | --- | --- | --- | --- | --- | --- | --- | --- | --- |
|  | **Aim 1** | | **Aim 2a** | | | | **Aim 1** | | **Aim 2a** | | | | |
|  | **ADHD persisters vs controls** | | **ADHD persisters vs remitters** | | **ADHD remitters vs controls** | | **ADHD persisters vs controls** | | **ADHD persisters vs remitters** | | **ADHD remitters vs controls** | | |
|  | ***β (95% CI)*** | ***p*** | ***β (95% CI)*** | ***p*** | ***β (95% CI)*** | ***p*** | ***β (95% CI)*** | ***p*** | ***β (95% CI)*** | ***p*** | ***β (95% CI)*** | ***p*** |  |
| **Mu** | 0.21 (-0.03; 0.45) | 0.092 | 0.12 (-0.26; 0.51) | 0.523 | 0.08 (-0.28; 0.45) | 0.654 | *0.43 (0.22; 0.67)* | <0.001** | *0.40 (-0.03*; 0.73) | 0.075 | 0.09 (-0.27; 0.46) | 0.614 |  |

**Collapsed across condition**

|  | | **Aim 1** | | **Aim 2a** | | | |
| --- | --- | --- | --- | --- | --- | --- | --- |
|  |  | **ADHD persisters vs controls** | | **ADHD persisters vs remitters** | | **ADHD remitters vs controls** | |
|  |  | ***β (95% CI)*** | ***p*** | ***β (95% CI)*** | ***p*** | ***β (95% CI)*** | ***p*** |
| **Sigma** | | *0.33 (0.10, 0.42)* | 0.003* | *0.32 (-0.01, 0.68)* | 0.060 | -0.01 (-0.34, 0.32) | 0.943 |
| **Tau** | | **0.69 (0.49; 0.89)** | <0.001** | *0.40 (0.07; 0.73)* | 0.016* | 0.19 (-0.02; 0.20) | 0.079 |
| **Theta ERSP** | FC | -0.28 (-0.44; -0.11) | <0.001** | -0.22 (-0.40; 0.04) | 0.065 | -0.04 (-0.30; 0.22) | 0.751 |
|  | CP | **-0.50 (-0.76; -0.24)** | <0.001** | *-0.37 (-0.75; -0.24)* | 0.022* | -0.12 (-0.49; 0.23) | 0.494 |
| **Theta ITC** | | *-0.42 (-0.47; -0.13)* | <0.001** | *-0.40 (-0.79; 0.01)* | 0.043* | -0.01 (-0.39; 0.35) | 0.925 |

Abbreviations: ADHD, attention-deficit/hyperactivity disorder; ERSP, event-related spectral perturbation; FC, fronto-central; CP, centro-parietal. Notes: For aim 1, the p-value threshold surviving multiple testing correction was determined as 0.003 using false discovery rates (FDR). Post-hoc tests are reported in by condition only for measures showing significant group-by-condition effects. For measures showing non-significant group-by-condition effects, post-hoc tests are reported across conditions. Ex-Gaussian variables were available for 86 persisters, 23 remitters, and 166 controls. ERSP and theta phase consistency variables were available for 81 persisters, 23 remitters, and 163 controls. **p<0.01, *p<0.05. Bold=large effect size (β ≥50); Italics=medium effects size (β≥. .30).

**Table S5**. Random-intercept linear models of ex-Gaussian and EEG time-frequency measures with parent-reported ADHD symptoms and impairment within the ADHD group only, controlling for age and sex and IQ (top half) and without covariate (bottom half)

| **Aim 2b** | | **ADHD symptoms** | | **Impairment** | |
| --- | --- | --- | --- | --- | --- |
|  | | ***β*** | ***p*** | ***β*** | ***p*** |
| **Covarying for sex, age, and IQ** | | | | | |
| **Mu** | | -0.07 (-0.36; 0.23) | 0.671 | - | - |
|  | Baseline | - | - | -0.09 (-0.27; 0.08) | 0.304 |
|  | Fast-incentive | - | - | 0.08 (0.09; 0.26) | 0.355 |
| **Sigma** | | 0.07 (-0.39; 0.54) | 0.766 | -0.12 (-0.65; 0.39) | 0.636 |
| **Tau** | | 0.22 (-0.09; 0.54) | 0.175 | 0.31 (0.04; 0.67) | 0.087 |
| **Theta phase consistency** | | 0.09 (-0.26; 0.45) | 0.611 | -0.06 (-0.47 -0.33) | 0.513 |
| **Without covariates** | | | | | |

| **Mu** | | -0.01 (-0.15; 0.21) | 0.751 | - | - |
| --- | --- | --- | --- | --- | --- |
|  | Baseline | - | - | -0.01 (-0.18; 0.17) | 0.943 |
|  | Fast-incentive | - | - | 0.22 (0.03; 0.41) | 0.019* |
| **Sigma** | | 0.07 (-0.35; 0.22) | 0.647 | -0.06 (-0.27; 0.39) | 0.740 |
| **Tau** | | 0.04 (-0.23; 0.14) | 0.645 | -0.09 (-0.29; 0.11) | 0.385 |
| **~~Theta ITC~~ Theta phase consistency** | | -0.03 (-0.26; 0.18) | 0.746 | -0.06 (-0.24; 0.33) | 0.994 |

Abbreviations: ADHD, attention-deficit/hyperactivity disorder; ERSP, event-related spectral perturbation.

Notes: Ex-Gaussian variables were available for 86 persisters, 23 remitters, and 166 controls. ERSP and theta phase consistency variables were available for 81 persisters, 23 remitters, and 163 controls. **p<0.010, *p<0.050. Bold=large effect size (β ≥.50); Italics=medium effects size (β ≥.30). Analyses of ADHD symptoms and impairment with all variables, as well as for mu with ADHD symptoms, were run collapsing across baseline and fast-incentive conditions, as the interactions with condition were non-significant (p>0.10).

**Table S6.** Associations of mu and tau with centro-parietal theta ERSP and theta phase consistency, with interactions between group (ADHD persisters, remitters, controls) and theta ERSP or theta phase consistency.

| Aim 3 | | Mu Baseline | | | | | | | Mu Fast-incentive | | | |  |
| --- | --- | --- | --- | --- | --- | --- | --- | --- | --- | --- | --- | --- | --- |
|  |  | **Main effect** | | | **Interaction** | | | | **Main effect** | | **Main effect** | |  |
|  |  | ***β*** | ***p*** | | ***β*** | | ***p*** | | ***β*** | ***p*** | ***β*** | ***p*** |  |
| Theta ERSP CP | | -0.22 (0.11; 0.33) | <0.001** | | -0.02 (-0.16; 0.10) | | 0.664 | | -0.32 (0.20; 0.43) | <0.001** | -0.06 (-0.06; 0.19) | 0.343 |  |
| Theta phase consistency | | -0.30 (-0.41; -0.20) | <0.001** | | -0.07 (-0.03; 0.19) | | 0.185 | | -0.32 (-0.46; -0.17) | <0.001** | -0.05 (-0.06; 0.18) | 0.374 |  |
| Aim 3 | | **Tau collapsed across conditions** | | | | | | |  | | | |  |
|  |  | **Main effect** | | | | **Interaction** | | |  | | | |  |
|  |  | ***β*** | | ***p*** | | ***β*** | | ***p*** |  | | | | |
| Theta ERSP CP |  | -0.10 (0.18; 0.02) | | <0.001** | | -0.03  (-0.11; 0.05) | | 0.479 |  | | | | |
| Theta phase consistency | | -0.31  (-0.39; -0.24) | | <0.001** | | -0.01(-0.08; 0.07) | | 0.881 |  |  |  |  |  |

Abbreviations: ERSP, event-related spectral perturbation; CP, centro-parietal. Abbreviations: ERSP, event-related spectral perturbation; CP, centro-parietal; ITC, inter-trial phase coherence. Notes: the p-value threshold surviving multiple testing correction was determined as 0.001 using false discovery rates (FDR). Ex-Gaussian variables were available for 86 persisters, 23 remitters, and 166 controls. ERSP and theta phase consistency variables were available for 81 persisters, 23 remitters, and 163 controls. Post-hoc tests examining the association between measures within each group were run only when the interaction with group was significant, thus for none of the variables. **p<0.01, *p<0.05. Bold=large effect size (β≥.50); Italics=medium effects size (β≥.30)

**
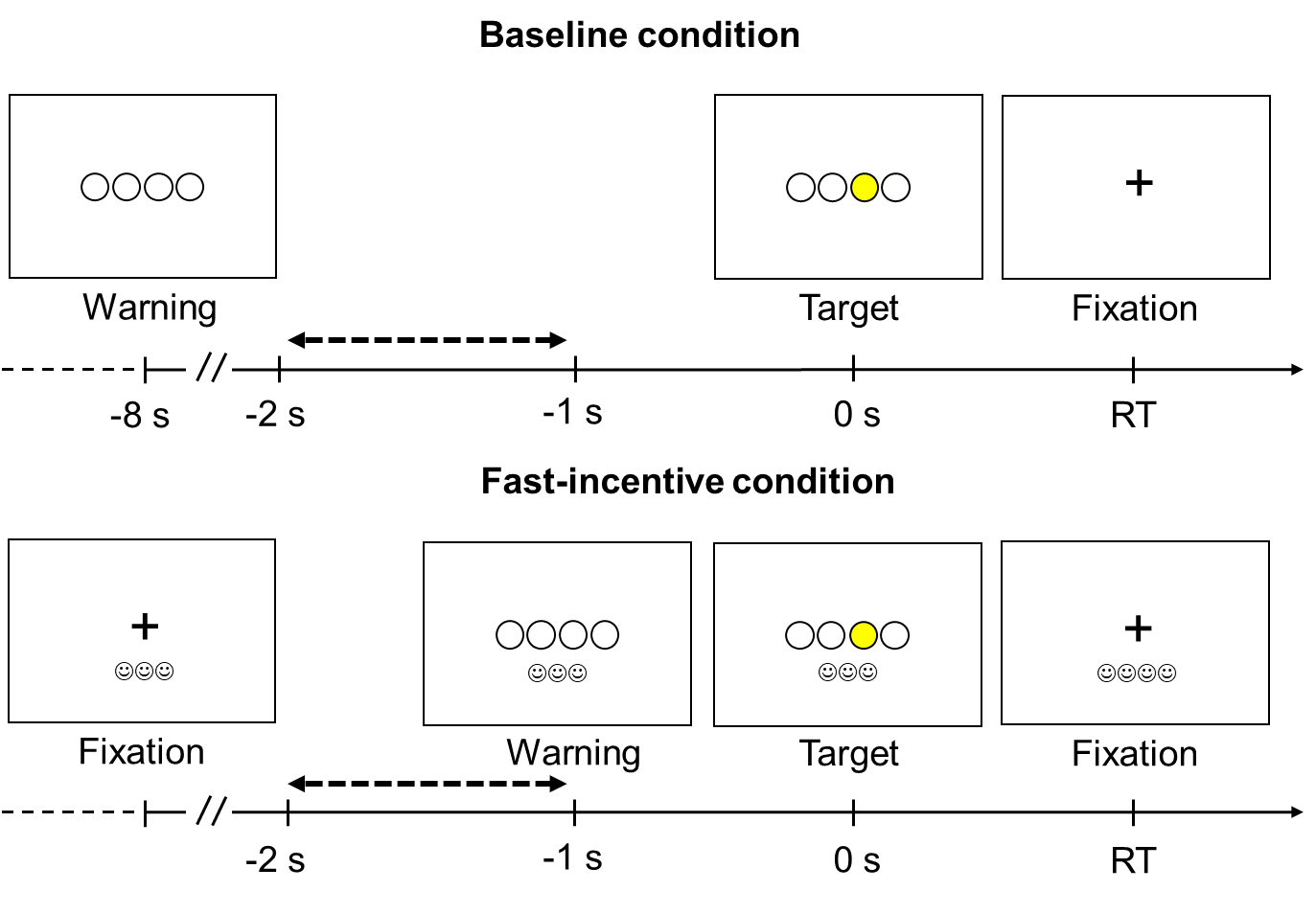
**

**Figure S1.** A schematic illustration of the temporal sequence of events in the baseline and fast‐incentive conditions of the Fast task. Notes: In both conditions, the warning remained on the screen until target onset. The target remained on the screen up to 10 s until a response (response time [RT]), followed by a fixed 2.5 s inter-trial interval. The double-headed dashed window corresponds to the pre-stimulus baseline window (-2 to -1 s) used to normalize the event-related spectral perturbations (ERSPs).


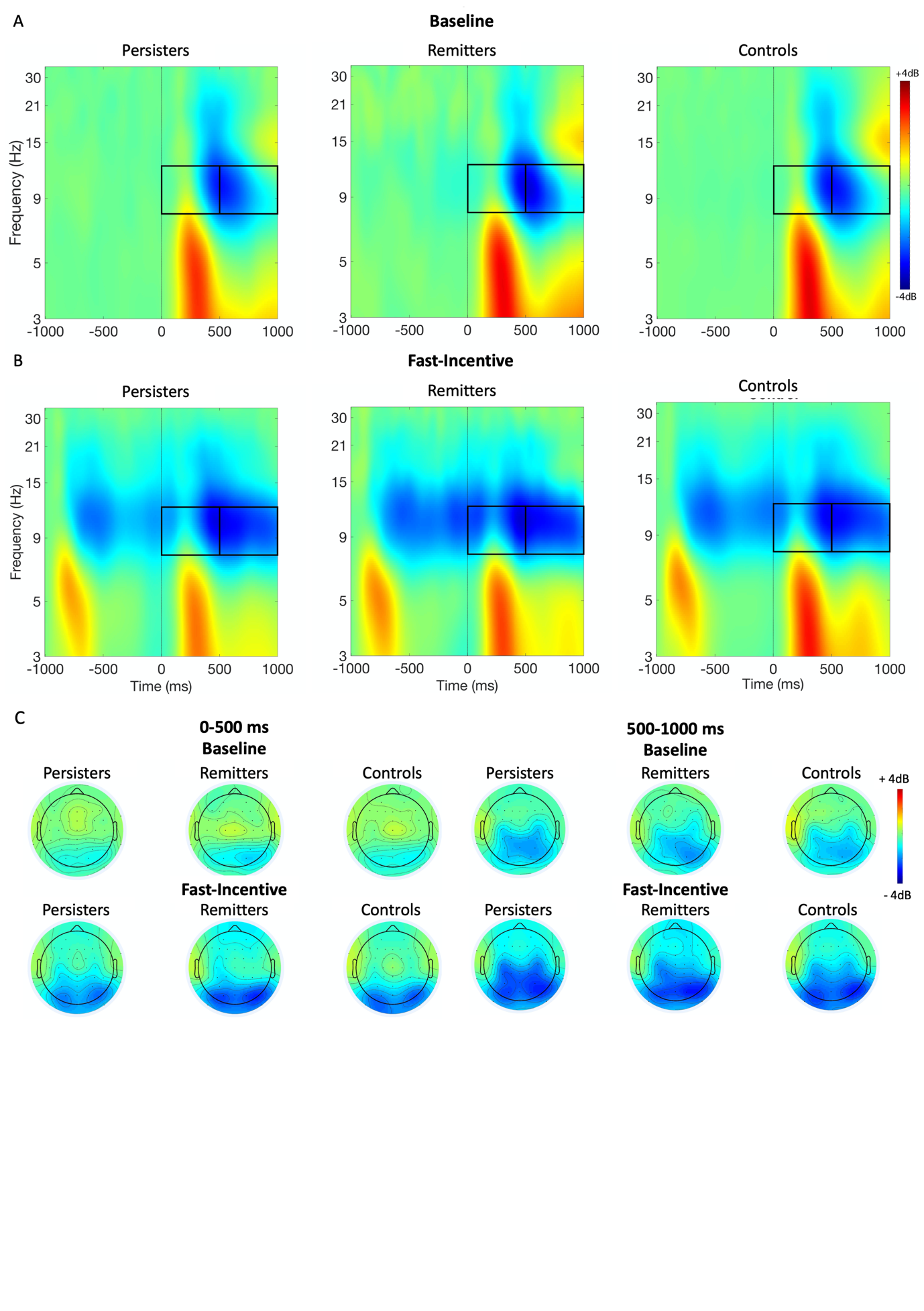


**Figure S2.** Alpha event-related spectral perturbation (ERSP) at parietooccipital regions in the ADHD persisters, ADHD remitters, and control groups in the baseline and fast-incentive condition of the Fast task. A. ERSP in the baseline conditions; B. ERSP in the fast-incentive condition; C. topographic maps by group in the 0-500 ms and 500-1000 ms windows at each condition.


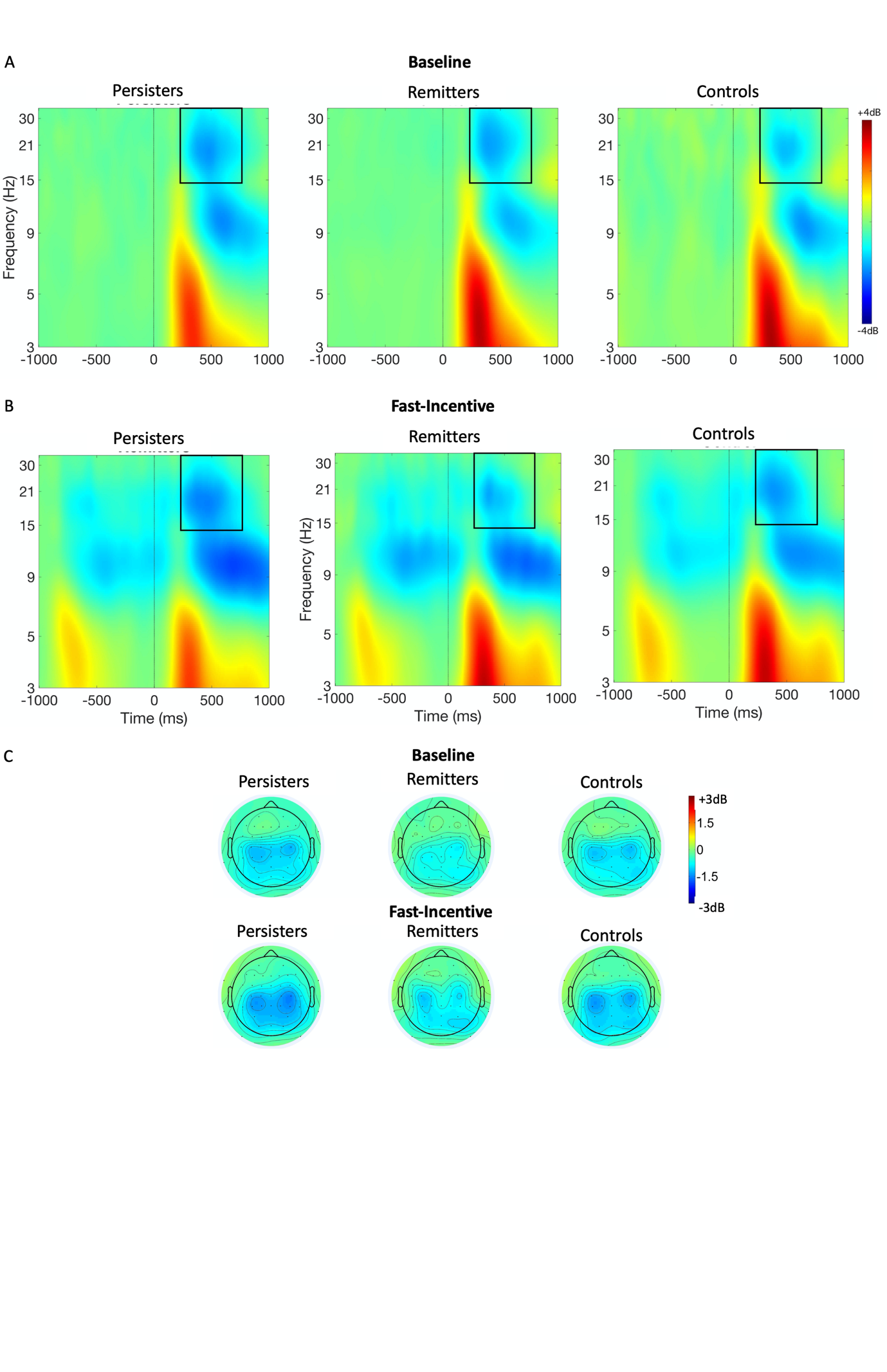


**Figure S3.** Beta event-related spectral perturbation (ERSP) at central regions in the ADHD persisters, ADHD remitters, and control groups in the baseline and fast-incentive conditions of the Fast task. A. ERSP in the baseline condition; B. ERSP in the fast-incentive condition; C. Topographic maps by group in the 200-700 ms.

**References**

**Cheung CHM, Rijdijk F, McLoughlin G, Faraone SV, Asherson P, & Kuntsi J** (2015). Childhood predictors of adolescent and young adult outcome in ADHD. *Journal of Psychiatric Research* **62**, 92–100.

**Delorme A, & Makeig S** (2004). EEGLAB: an open source toolbox for analysis of single-trial EEG dynamics including independent component analysis. *Journal of Neuroscience Methods* **134**, 9–21.

**Makeig S, Debener S, Onton J, & Delorme A** (2004). Mining event-related brain dynamics. *Trends in Cognitive Sciences* **8**, 204–210.

**Michelini G, Kitsune V, Vainieri I, Hosang GM, Brandeis D, Asherson P, & Kuntsi J** (2018b). Shared and Disorder-Specific Event-Related Brain Oscillatory Markers of Attentional Dysfunction in ADHD and Bipolar Disorder. *Brain Topography* **31**, 672–689.

**Tallon-Baudry C, Bertrand O, Delpuech C, & Pernier J** (1996). Stimulus specificity of phase-locked and non-phase-locked 40 Hz visual responses in human. *The Journal of Neuroscience* **16**, 4240–4249.
